# Supplementary material for: A Qualitative Study on Engaged Families’ Experiences with Long-Term Follow-Up Care in the Colorado/Wyoming Newborn Screening System
Source: Int J Neonatal Screen. 2024 Sep 11;10(3):61. doi: 10.3390/ijns10030061 (PMC11417874; doi:10.3390/ijns10030061)
Supplement: Supplementary file 1 [file IJNS-10-00061-s001.zip › IJNS-3136921-supplementary.pdf]

Focus Group Guide

[Introduction read by facilitator]

Thank you for joining us today. My name is _____ and this is my colleague _____. We are working on a grant project that is looking at long-term follow-up care for families who have at least one child with a condition that was diagnosed after a positive/abnormal newborn screen. As part of this project, we are gathering input from families and providers on what they think long-term follow-up care or support for families should look like. We will share what we learn with specialty care clinics in Colorado, Wyoming, and national programs interested in long-term follow-up. Our goal is to make sure families' voices are part of the discussion around what long-term follow-up care should look like.

We will not mention anyone by name in any reports or presentations we share. We will share general themes. If we provide a quote, we will make sure there is no identifying information in that quote.

Please be as honest as possible. Please also respect that others may have had different experiences or want different outcomes. We welcome different viewpoints as it provides a more complete picture for us. Also, please know that you do not have to answer any question you do not want to answer.

This focus group will last no more than 1.5 hours. At the end, we have a short survey for you to fill out so we can capture which groups we have talked with.

Finally, we'd like to record this so we can accurately capture your thoughts and ideas. No one outside the research team will hear the recording. Are you all okay with us recording this?

Please note, when we ask about "your child" in the following questions, if we don't specify otherwise, we are referring to your child who was diagnosed with a genetic condition after a positive/abnormal newborn screen.

Before we start, can each of you tell us your first name, how many children you have at home, and what genetic condition your child or children have that are being seen by Children's?

1. In your view, how would we know if a family whose child was identified by a positive/abnormal newborn screen is getting the care they need after a diagnosis?
    a. What would be happening for that family?
    b. What would not be happening?

2. What makes it hard for families to get the level of healthcare they want for their child who has a genetic condition diagnosed in infancy?
    a. What makes it easy to get the care?
    b. Why do you think some families aren't engaged in the healthcare system even though their child has a genetic condition diagnosed in infancy?

3. Is there anything that would improve the care you want and need for your child?

4. How often do you think your child should be seen in a specialty clinic?

5. What do you do if you disagree with what your child's doctors are saying?

6. If you have multiple doctors for your child, how well do they communicate to care for your child?
    a. What do they do well?
    b. What do you wish they would do that they don't?
    c. What do you wish they would do differently?

7. What are your goals for your child? [goals don't have to be related to healthcare]
    a. Probe along the lines of HRSA's CYSHCN Blueprint: that they are integrated in school, able to do school sports, eventually get a job, maybe own a home, etc. → life is more than a series of medical care.
